# Learning to prescribe intravenous fluids: A scoping review

**DOI:** 10.1007/s40037-017-0386-5

**Published:** 2017-11-08

**Authors:** Richard F. R. McCrory, Gerard Joseph Gormley, Alexander Peter Maxwell, Tim Dornan

**Affiliations:** 1 Centre for Medical Education, Queens University Belfast, Belfast, Northern Ireland, UK; 2 Regional Nephrology Unit, Belfast City Hospital, Belfast, Northern Ireland, UK

**Keywords:** Intravenous fluids, Prescribing, Medical education

## Abstract

**Introduction:**

Prescribing intravenous (IV) fluid therapy is a core skill expected of qualified doctors at the point of graduation, but medical graduates often feel ill-equipped to perform this task. This lack of preparedness contributes to treatment-related patient harm. This scoping review maps the current state of published evidence about how junior doctors prescribe IV fluid therapy and learn how to do it.

**Methods:**

We searched five electronic databases and grey literature from 1994 until June 2016 for articles describing any aspect of IV fluid prescribing practice or its education. A total of 63 articles were selected for analysis. Using the WHO Guide to Good Prescribing to categorize the extracted findings, our review focuses on prescribing IV fluids in adult generalist settings.

**Results:**

Most articles studied IV fluid prescribing from the perspective of the doctor. Junior clinicians struggled to conceptualize IV fluid prescribing as a ‘whole task’ in authentic work settings and lacked support. Educational interventions to improve IV fluid prescribing often focused on enhancing prescriber knowledge about fluid and electrolyte balance rather than execution of the prescribing task.

**Conclusions:**

Our understanding of IV fluid prescribing as a holistic integrated skill is patchy, as is its performance. Current IV fluid prescribing education appears insufficient to foster safe and effective practice. For education to achieve the ultimate goal of safer prescribing in workplaces, we need a clearer understanding of how healthcare professionals prescribe IV fluids in real world practice.

**Electronic supplementary material:**

The online version of this article (10.1007/s40037-017-0386-5) contains supplementary material, which is available to authorized users.

## What this paper adds

Novice clinicians are responsible for most prescribing of intravenous (IV) fluids in hospital settings. Ensuring they are ready to perform this skill safely is a necessary concern for educators and practitioners. Understanding how doctors prescribe IV fluids and learn how to do it will aid the preparation of learners, but the extent of evidence describing such practices is not known. This exploratory literature synthesis describes the activities doctors employ while prescribing IV fluids, summarizes the nature of educational initiatives implemented to improve fluid prescription quality and proposes topics for future educational research and development.

## Introduction

Prescribing intravenous (IV) fluids is one of the first and most frequent tasks a newly qualified doctor is expected to do [1, 2]. Thus, relatively inexperienced clinical trainees prescribe the majority of IV fluids outside operating theatres and critical care environments. Some patients, unable to take oral fluids but otherwise stable, need maintenance IV fluid therapy, others have rapidly evolving fluid and electrolyte requirements. The junior doctors who manage this range of clinical situations prescribe IV fluid therapy under limited supervision. New graduates, however, find their training has poorly prepared them for this task [2, 3]. The UK National Institute for Health and Care Excellence (NICE), recognizing this lack of readiness as a risk to patients, made IV fluid therapy an educational priority [4]. Preparing new graduates to prescribe IV fluids is, therefore, of urgent concern to patients, practitioners, educators and those responsible for patient safety.

While many authorities regard IV fluids as drugs [5, 6] and assessments of prescribing competence [7, 8], such as the UK Prescribing Safety Assessment, can include questions on IV fluid therapy, educators have not always afforded them the same status as other medicines. Systematic reviews of education to improve prescribing have found no research into IV fluid therapy [9–11]. IV fluids are notable by their absence from a compilation of medicines that are core to prescribing education [12]. The reason for this disparity is not entirely apparent.

Prescribing assessments alone, however, do not guarantee safe practice. Earlier research has shown that prescribing is embedded in social contexts that make the performance of apparently simple tasks complex and error-prone [13–17]. Prescribing IV fluids, moreover, is not a ‘one-off’ task but a dynamic, evolving process, of which monitoring patients’ responses to the administered agent is an integral part. We regard ‘prescribing’ as an inherently complex ‘whole task’ [14], which includes assessing patients’ needs in context, assessing factors intrinsic and extrinsic to patients that complicate treatment choices, administering or delegating administration of treatment, assessing how all those variables change over time, and judiciously tailoring therapy to take account of those factors. Current training and testing of prescribing, however, focuses on these elements of the task in a disjointed way, usually outside a clinical context [14, 18].

Understanding how doctors prescribe IV fluids and learn to do so could improve their preparation for practice and the quality of prescriptions once they graduate. Since there has been no review of this topic, our aim was to identify all relevant publications, draw whatever conclusions the evidence could support, and identify topics for future educational research and development. We chose to undertake a scoping review because this methodology is exploratory in nature, more inclusive than others, and aims to map current evidence relevant to a topic.

## Methods

The review followed the methodological steps for scoping reviews devised by Arksey and O’Malley [19] as revised by Levac and colleagues [20, 21].

### Ethics

Research ethics approval was not required because this was a secondary analysis of literature within the public domain and no subjects participated.

### Research team

The research team comprised a senior specialist trainee in renal medicine (RFRM), and three physicians (a general practitioner (GJG), a nephrologist (APM) and endocrinologist (TD)) involved in educational research and/or clinical teaching.

### Step 1: Identifying the research question

Healthcare professionals typically learn through practice in workplace environments. This experiential learning in clinical settings makes significant contributions to learners’ development of their skills, such as prescribing IV fluids. It is essential that the circumstances of prescribing preparation closely align with what those in practice are expected to know, do and value [22]. We wanted to explore both prescribing and ways of learning to prescribe because we reasoned that educational conclusions require anchoring in a clear understanding of the task in practice.

While junior doctors prescribe most IV fluids, and do so ordinarily in general medical and surgical settings, we began with a very inclusive, exploratory question to obtain as full information as possible about IV fluid prescribing by anyone in any context: ‘What is known about how health professionals prescribe intravenous fluid therapy, and how they learn to do so?’

### Step 2: Finding relevant articles

We interrogated five electronic databases, specifically: PUBMED/MEDLINE, EMBASE, CINAHL, SCOPUS, and Web of Science. The search strategy was based on search terms originally used by the NICE guideline CG174 [4], with modifications. The MEDLINE search strategy is shown in Table 1. A subject librarian with expertise in medical databases helped adapt the search terms for each archive (The Literature Search Strategies are available in online Electronic Supplementary Material, Document A). Scoping reviews typically include the examination of full-text grey literature sources [19] (including theses, reports, working papers, government documents, white papers, and evaluations) so we used keywords from the MEDLINE strategy to do a title-only search in Google & Google Scholar (Google Inc., Mountain View, USA). Citation lists were organized using Paperpile Software (Paperpile LCC, Vienna, Austria).

**Table 1:** MEDLINE search terms

1. clinical competence/
2. health knowledge, attitudes, practice/
3. physician’s practice patterns/or nurse’s practice patterns/
4. (train* or educat* or teach* or apprais* or learn*).ti,ab
5. (knowledge or attitude*).ti,ab
6. (perception* or opinion* or responsibilit*).ti,ab
7. ((core or clinical or key or complex) adj2 skill*).ti,ab
8. (profession* adj2 develop*).ti,ab
9. (audit or (qual* adj2 improv*)).ti,ab
10. fluid therapy/
11. water-electrolyte balance/
12. ((fluid* or volum*) adj3 (therap* or intravenous* or iv or infusion* or drip* or administrat*)).ti,ab
13. ((fluid* or volum*) adj3 (restor* or resuscita* or replac* or deplet* or deficien*)).ti,ab
14. (fluid* adj3 (challenge or bolus)).ti,ab
15. ((crystalloid* or colloid*) adj3 (therap* or intravenous* or iv or infusion* or drip* or administrat*)).ti,ab
16. ((fluid* or volum*) adj3 (balance* or imbalance* or manag* or maint* or loss* or status or monit* or assess* or reassess* or evaluat* or prescri* or document* or chart* or protocol or strateg* or regimen* or require* or need*)).ti,ab
17. 1 or 2 or 3 or 4 or 5 or 6 or 7 or 8 or 9
18. 10 or 11 or 12 or 13 or 14 or 15 or 16
19. Hospitals.mp. or hospital/ or (hospital* adj3 staff*).mp
20. 17 and 18 and 19
21. limit 20 to (English language and humans and yr=“1995–2016”)

To strike a balance between evidence that remained relevant to current clinical practice, and ensuring a pertinent time period to capture changes in medical education and practice after the release of the General Medical Council’s ‘Tomorrow’s Doctors’, the search was limited to between January 1994 and June 2016.

### Step 3: Selection of relevant articles

After removing duplicates, the first author (RFRM) screened all abstracts retrieved by the search and rejected those that were not relevant to the practice of IV fluid therapy or any aspect of its education. The whole research team convened at this stage to assess whether the search strategy was identifying evidence sources applicable to the topic under review. We excluded 42 articles that were only available as a conference abstract, as is customary in scoping review methods. At this stage, we identified no articles exploring fluid prescribing from the perspective of pharmacists in practice. We only, therefore, describe studies that represent doctors, nurses, or students of these professions prescribing, administering, or monitoring IV fluid therapy to adult patients in hospital settings. Two authors (RFRM and GJG) independently reviewed full-text copies of all screened papers to determine their inclusion in the analysis, based on the criteria described in Table 2. The team continued to work collaboratively throughout the review, evaluating how the data were charted and collated.

**Table 2:** Eligibility criteria for paper selection

**Inclusion criteria were any full-text published work that describes some or all of the following:**
– Participants of the studies were doctors, nurses, or students of these professions – Studies examined the activities involved in prescribing, administering, or monitoring IV fluid therapy to adult patients – Studies examined efforts to educate health professionals or students to prescribe, administer or monitor IV fluid therapy in adults – Studies involved IV fluid therapy provided in a hospital setting
**Exclusion criteria**
– Studies involving other fluids (such as IV antibiotics and parenteral nutrition), fluids by other routes (subcutaneous or oral) or transfusion of blood products – Clinical trials comparing regimens of IV fluid therapy – Expert review or commentary articles on prescribing IV fluids – Studies of IV fluid prescribing in outpatient or nursing home settings – Studies of IV fluid prescribing in paediatric, emergency or critical care, or other non-generalist settings

### Step 4: Charting the data

**Fig. 1:**
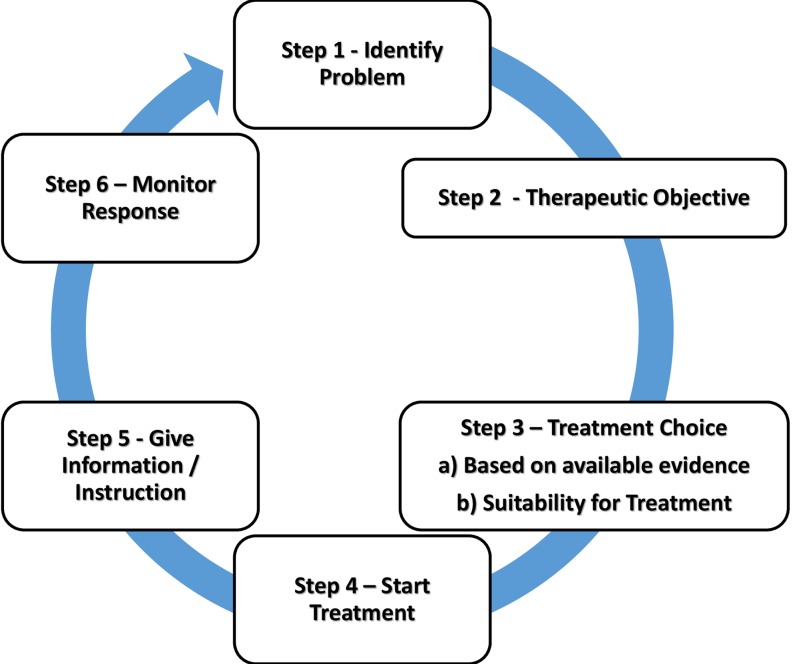
Steps in the WHO Good Prescribing Guide (from Dr Vries et al. [23])

RFRM created a data extraction sheet using Microsoft Excel (Microsoft, Redmond, USA), and populated it with demographic and methodological details. We used the WHO Guide to Good Prescribing ([23]; Fig. 1) as a framework to categorize the prescribing activities described in the source articles and organize the findings. This model encourages prescribers to apply a systematic ‘whole-task’ approach to individual patient requirements when choosing a drug and has the largest body of evidence supporting its use in improving prescribing competencies internationally [11].

### Step 5: Collating and summarizing the data

**Fig. 2:**
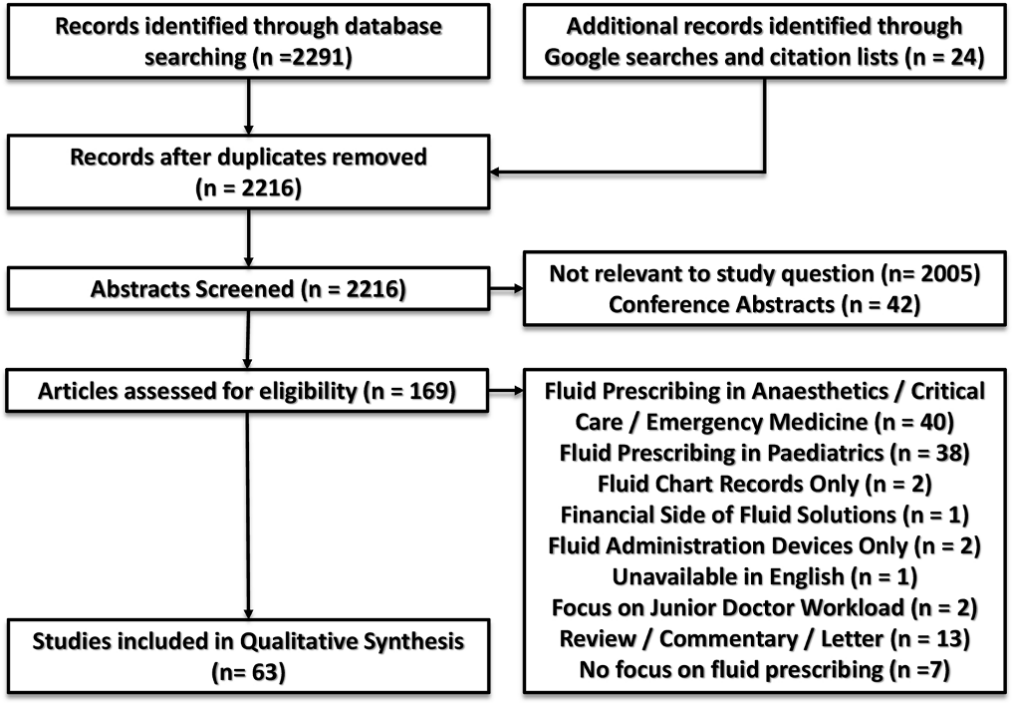
Study flow diagram indicating the number of records identified, included, and excluded, with reasons for exclusion

The Preferred Reporting Items for Systematic Reviews and Meta-Analyses (PRISMA) [24] criteria and guidelines from the Joanna Briggs Institute [25] guided the conduct and reporting of the review. A flowchart of the selection process, with reasons for excluding papers, is shown in Fig. 2. The heterogeneity of research data precluded a quantitative analysis, so we used a qualitative approach to collate, summarize, and map the data.

## Results

### Origins and dates of publication

Of the 63 articles selected for this analysis, 45 (71%) originated from the United Kingdom, four from Canada, three each from Australia and the USA, two from New Zealand, and one each from China, Iran, Kuwait, Sudan, the Netherlands and Turkey. Forty-four (70%) articles were published after 2008, the year of publication of the first UK guidelines on IV fluid prescribing [26]. Forty-nine (78%) articles reported how IV fluids were prescribed and 14 described how people learned to prescribe them. Educational methods included didactic teaching, an audited multidisciplinary intervention, and comparisons against a guideline. The majority of articles, 35 (56%), studied doctors’ IV fluid prescribing practice alone. Multidisciplinary teams were studied in 19 (30%) of the papers, though the authors restricted their attention to physicians in these groups. Five references (8%) examined the delivery of IV fluid therapy from the perspective of nurses, and four (6%) focussed on medical students.

### Identifying a need for treatment (corresponds to steps 1–2 of the WHO guide)

Sources of information were direct observations of practice and questionnaires. Most participants were junior doctors or medical students. Outside normal working hours, the junior doctors were usually unfamiliar with the patients they prescribed IV fluids for [27–29]. They infrequently examined patients and, when they did, could not reliably determine fluid volume status [27]. Discussing patients with nurses, reviewing laboratory results, and reading medical records and fluid balance charts contributed to the assessment of clinical need [28, 30] though junior doctors did not always interpret those sources of information correctly [31].

### Choosing an IV fluid therapy regimen (Steps 3–4)

#### Variations in treatment initiation

The widely varying indications for starting IV fluid therapy [32] and differences between junior and senior clinicians’ treatment criteria [29, 32, 33] suggest that junior doctors were delegated the IV fluid prescribing task but were not closely supervised [2, 34]. Supervision was made more significant by variations between clinical specialties and contexts of care in the volumes and types of IV fluid recommended for different conditions [33, 35–37]. Prescribers did not always consider a patient’s body weight, fluid volume, or electrolyte status [2, 33, 37–44].

#### Rules and guidelines

Both informally shared rules and formal guidelines influenced IV fluid prescribing. It was common practice, for example, to calculate maintenance IV fluid requirements by adding insensible losses to the previous day’s urine output [2]. It was uncommon, however, to take the patient’s body weight into account [37, 45]. A questionnaire survey using case vignettes showed that, contrary to current guidance for IV fluid resuscitation in sepsis [46], junior doctors’ choices of IV fluid volumes were unrelated to body weight. In some instances, junior doctors were unaware of available guidance or struggled to reconcile differences between directives [2, 29, 47].

### Evaluate comorbidity (Step 3b)

Textbooks usually describe IV fluid regimens for stable, healthy patients [48, 49] whereas many patients, particularly those admitted urgently to surgical and medical wards, have complicating comorbid conditions [38, 41, 50–53]. Few articles examined the relationship between comorbidity and adverse outcomes. One exception was a retrospective survey of patients admitted with hypernatraemia. Approximately a fifth of these patients did not receive treatment because they were terminally ill and IV fluid therapy was thought inappropriate [54]. Those who received IV fluids in this series had a high level of physical dependence, dementia, and other life-limiting illnesses. Adhering to local guidelines rarely corrected their serum sodium at an appropriate rate and over half of patients died during that admission [54].

### Communication (Step 5)

The articles contained no information about what patients are asked or told. Clinical records documented the treatment indications and regimen inconsistently [29, 50, 53, 55–57] although there were isolated examples of excellence [58].

### Monitoring treatment response (Step 6)

Neither in maintenance IV fluid therapy nor IV fluid resuscitation were there any reports of how clinicians evaluated responses to treatment. There was, however, evidence from the treatment of sepsis in a National Confidential Enquiry into Patient Outcome and Death (NCEPOD) report, where it was insufficient in up to a third of cases [50]. Staff often inserted urinary catheters into frail patients in surgical wards [30] to monitor their urine output but were less likely to track such patients’ biochemical responses and body weight than in less infirm patients [30].

### Learning IV fluid therapy

#### Using education to improve IV fluid prescribing knowledge

Despite a perceived lack of teaching about IV fluid therapy, most junior doctors and medical students regarded their ability to prescribe IV fluid therapy as acceptable, and judged themselves aware of its potential hazards [1, 27, 32, 47, 59]. Foundation knowledge of biochemistry and physiology underpinning IV fluid prescribing, however, varied widely between educational institutions, specialties, and levels of experience [2, 29, 47, 59–63]. Education to improve IV fluid prescribing was primarily didactic teaching to resolve these deficiencies [29, 40, 61, 64–66]. While test scores improved and self-reported practice in the short term increased, there was no information about its longer-term effects on knowledge or clinical performance. There were large differences in the treatment regimens and ways of estimating IV fluid requirements that different textbooks and guidelines recommended [48, 49].

#### Using education to improve IV fluid prescribing performance

Multidisciplinary teams were educated to follow protocols [55, 67–70], whose rules included paying attention to the patient’s body weight, assessing ongoing fluid requirements, communicating with other team members in writing [70], and matching IV fluid regimens to predicted fluid losses [68]. Tools to reinforce these rules included algorithms on posters, online guidelines, and care bundles specific to IV fluid therapy.

These interventions improved elements of IV fluid therapy that were performed well but had less impact on elements executed poorly [55, 67]. Better recognition of acute kidney injury associated with hypotension or volume depletion, for example, did not result in more appropriate resuscitation with IV fluids [55]. In contrast, educating clinicians to prescribe fluid regimens that were relevant to patients’ needs increased prescribing performance in test hospitals compared with controls [68]. Nurses trained to follow guidelines on fluid balance recording improved performance in this area [70]. Additionally, nurses following protocols to guide fluid replacement for gastric and intestinal losses ordered slightly more appropriate IV fluid regimens [68] although, as with didactic teaching, effects on clinical endpoints were not recorded.

There have also been educational quality improvement projects targeting completion of fluid balance charts and prescriptions, particularly in out-of-hours settings, where junior doctors were poorly supervised [71–75]. The transferability of these findings was limited by being very context-specific, influenced by local variations in practice, and lacking long-term follow-up.

### Effects of workplace learning environments on performance

Researchers have examined how different learning environments influence monitoring and prescribing of IV fluid therapy. Although fluid balance and observation charts were acknowledged to be essential to good prescribing [29, 57, 76], time constraints, pressure to complete other tasks, and insufficient staffing [29, 56, 57, 76] resulted in these being inaccurate or incompletely completed. Different supervising clinicians may give junior doctors and medical students conflicting advice [3, 29].

### Errors in IV fluid prescriptions and their consequences

The types of error described in the articles broadly grouped into ordering appropriate IV fluid prescriptions too late [50, 51, 55, 77], ordering inappropriate IV fluid volumes, types, or rates of administration [43, 44] or incomplete documentation of the fluid prescription [43]. Prescribers provided insufficient water to patients with hyperosmolar dehydration [77–79], failed to anticipate falls in serum potassium in fasting diabetic patients on insulin infusions [80], and prescribed volume-expanding fluids for patients with a diagnosis of heart failure on admission [81].

Suboptimal IV fluid therapy, characterized by either excessive or inadequate replacement of fluid volume, often related to insufficient record-keeping and was a persistent problem [38, 41, 50–53, 77]. Inconsistent data collection stood in the way of examining the effect of poor practice on patient outcomes [38, 50, 51, 53]. Patients in general surgical wards with systemic disease or functional limitations were at increased risk of complications of IV fluid or electrolyte therapy, particularly when they received higher volumes of IV fluid [82, 83]. Hospital-acquired hypernatraemia, which results from giving insufficient electrolyte-free water, increased the risk of morbidity and death [78, 79]. Patients with heart failure who received inappropriate IV fluids at admission had a higher likelihood of needing critical care support or dying during admission [81]. Mousavi et al. remarked that over 20% of IV fluid prescribing errors had the potential to cause harm, require intervention, prolong hospitalization, or, rarely, result in a patient’s death [44].

## Discussion

### Principal findings and meaning

The principal finding from our review is that novice clinicians struggled to practise the ‘whole task’ of prescribing IV fluids; they neglected some important aspects and did others incorrectly. Furthermore, they integrated mutually dependent parts of the task poorly. Available evidence perhaps offers clues to explain this. Junior doctors lacked skills to identify a need for IV fluids, particularly in patients who were unknown to them; these assessments occurred outside regular working hours, so senior help was probably not readily available. In the absence of supervision, clinicians prescribed IV fluids based on shortcuts learnt ‘on the job’, rather than relying on the findings of a physical examination, relevant investigations, or following formal guidelines. An over-reliance on such prescribing strategies, however, may have resulted in doctors overlooking specific aspects of patients’ needs assessments or comorbidity, especially in people admitted acutely to busy hospital environments. Support when available from senior colleagues or experts—whose practices stemmed from their historical workplace experiences and clinical specialty exposure—was inconsistent. This perpetuated broad differences in IV fluid prescribing behaviour.

Errors such as delayed treatment or misprescribed IV fluids were extreme examples of such variation in performance. The knowledge or skills of individual practitioners, though important, were not the only factors causing harm as described in national accounts of practice, such as the National Confidential Enquiry into Patient Outcome and Death report. Rather, an interplay of factors in workplace environments, including work demands, quality of documentation, interprofessional communication, and working relationships, contributed to mistakes.

These findings have ramifications for safe IV fluid prescribing and its education. In light of the above findings, the optimal conditions for novice prescribers acquiring proficiency in prescribing IV fluids appear contingent upon a holistic, integrated understanding of the task, exposure to workplace practices that enable its enactment in authentic contexts, and access to supportive feedback specific to individual learner needs. Novice doctors, furthermore, must also be able to negotiate the uncertainties created by the physiological variation in the patients they encounter and attend to differences in prescribing behaviour between groups of practitioners. This ability to adapt to differing requirements in work settings is itself an intellectual feat.

Educational interventions for IV fluid prescribing, in contrast, principally addressed deficiencies in the conceptual knowledge of IV fluid therapy or adherence to clinical guidelines. While factual capacities are readily amenable to assessment in educational settings, development of these attributes alone appears insufficient to foster a readiness to prescribe IV fluids safely in workplace environments. Novice doctors prescribed IV fluids repetitively—enabling rehearsal of skills we consider essential to IV fluid prescribing behaviour—but did so under limited supervision, so it is uncertain whether they possessed dispositional qualities to recognize these events as opportunities for learning, or had access to meaningful feedback on the outcomes of their actions. We believe that feedback on performance is critical in developing prescribers that can distinguish between IV fluid prescriptions that are straightforward and more challenging cases requiring effortful thought or more expedient senior help. Educators or practitioners must, therefore, begin to attend to the configuration of workplace (especially supervisory) relationships in health teams, which will be influential to the success of future interventions to improve IV fluid prescribing education, and ultimately, patient outcomes.

### Limitations

Our review focused on the single most commonly performed duty of newly qualified doctors, the poor performance of which can have potentially lethal consequences. Such specificity on adult intravenous fluid therapy, admittedly, limits the transferability of our findings. IV fluids are distinctive for their powerful, immediate effect on core physiology and biochemistry, as exemplified by their use in the resuscitation of critically ill patients. A prescriber cannot wholly predict in advance the effect of any drug in such a dynamic situation. Responsible prescribing, therefore, means evaluating probabilities of benefit and harm, making empirical choices, and carefully monitoring effects. IV fluids, therefore, are very similar to agents such as rapid-acting insulin and antiarrhythmic drugs. At the other extreme, prescribing anti-tuberculous therapy acts on timescales of months rather than minutes. Variables are more clearly defined, probabilities more easily calculated, and empirical outcomes evolve far more slowly. Readers should take these important differences into account and regard prescribing as a practice with many variations.

The exploratory nature of the scoping review methodology allowed us to include sources with a broad mix of study type and quality not ordinarily included in other syntheses. Some research on IV fluid therapy practices or its education was only published as abstracts, which did not fulfil our eligibility criteria; this may have limited the extent to which our sources comprise an authentic representation of reality, as is typical of any literature review. We invite readers from within the health professions to judge for themselves how far the findings of this study reflect the ways fluid prescribing and its education happen in similar settings.

Our decision to focus on IV fluid prescribing in adult generalist settings necessarily resulted in the exclusion of subsets of the literature that explore IV fluid therapy elsewhere, specifically paediatrics and critical care. There are notable differences between children and adults, principally related to physiological responses to fluid, and the need to prescribe as guided by weight. The practices underpinning IV fluid prescribing in children follow historical assumptions that current evidence now challenges. Similarly, in critical care settings, there is no compelling evidence demonstrating a ‘default’ IV fluid therapy for every clinical situation or determining an ‘optimal’ fluid resuscitation strategy. Our decision, however, makes the problem relevant to where novice doctors begin work and, as we have seen, do so with variable supervision. Additional research could examine how these specialist areas approach IV fluid prescribing and whether insights are transferable to prescribing for adults in non-critical settings.

The strength of using the Guide to Good Prescribing was that it provided a widely accepted framework to structure the findings of our review in a meaningful way. Its focus on the decision making between an individual prescriber and patient, however, likely underplays the relevance of cultural and organizational aspects we attempted to describe. This issue reflects the nature of the literature landscape we identified which emphasized behaviours of the prescriber and limited our capacity to comment on the contributions of other health professions (including nurses and pharmacists) to the co-ordination of IV fluid therapy or the contextual factors in the everyday interactions of work.

The predominance of articles from the United Kingdom likely exhibits an increased national awareness of the risks of IV fluids illuminated by national reports examining the quality of health delivery in hospital settings and an ongoing public inquiry into the deaths of children from cerebral oedema secondary to hyponatraemia following administration of intravenous hypotonic saline. Government departments responded to these events by developing guidelines through expert consensus on what good practice in IV fluid therapy looks like and addressing supposed insufficiencies in preparing health professionals for this task. We hope that identifying research areas of future interest through the scoping review process opens new avenues for investigation and practice globally into IV fluid therapy and its education.

### Implications for future work

Our study found a dearth of robust evidence on how clinicians prescribe IV fluids in practice and learn how to do it. We found gaps in the IV fluid prescribing literature, chiefly how clinicians monitor, evaluate, and adapt their practices. We also noted an absence of evidence describing the extent of patient involvement in prescribing decisions. Our appreciation of the ‘whole task’ remains, therefore, incomplete, and opens up an important research agenda.

Our insights into the many variables that affect the quality of IV fluid prescribing and its education imply that practitioners and educators should, first, acknowledge its inherent complexity. While the literature focused on junior doctors instead of the multidisciplinary team, it is important to recognize that several health professionals coordinate IV fluid therapy delivery, including nurses, and, to a variable extent, pharmacists. As a precursor to designing interventions to improve IV fluid prescribing performance and minimize treatment harm, it seems imperative to both evaluate how IV fluid prescribing in a real-world clinical environment happens and understand the influence of other disciplines in its delivery. Our next study will attempt to illustrate this complexity by observing instances of IV fluid therapy prescribing in everyday practice and learn how clinical teams, as a collective, reach this goal. Our research will also pay attention to the administration and monitoring of IV fluids, as these activities are fundamentally related to prescribing acts and the wider goals of patient care.

## Conclusions

Novice clinicians prescribe most IV fluid therapy, yet it is an incompletely understood and imperfectly performed task. Most IV fluid prescribing education is divorced from the messiness and complexity of real-world contexts and limited in its focus. Junior doctors, therefore, are underprepared to practise the ‘whole task’ in a contextually sensitive way. Practitioners and educators wanting to improve this situation must obtain a clearer understanding of IV fluid prescribing as an integrated, applied skill in workplace settings to inform this process.

## Caption Electronic Supplementary Material

Document A: Literature Search Strategies for the remaining four academic databases reviewed
