# Supplementary material for: Learning to prescribe intravenous fluids: A scoping review
Source: Perspect Med Educ. 2017 Nov 8;6(6):369–79. doi: 10.1007/s40037-017-0386-5 (PMC5732109; doi:10.1007/s40037-017-0386-5)
Supplement: Supplementary file 1 — Document A: Literature Search Strategies for the remaining four academic databases reviewed [file 40037_2017_386_MOESM1_ESM.docx]

Supplementary Document A: Literature Search Strategies

**A1 MEDLINE Search Terms**

| 1. clinical competence/ |
| --- |
| 2. health knowledge, attitudes, practice/ |
| 3. physician's practice patterns/ or nurse's practice patterns/ |
| 4. (train* or educat* or teach* or apprais* or learn*).ti,ab. |
| 5. (knowledge or attitude*).ti,ab. |
| 6. (perception* or opinion* or responsibilit*).ti,ab. |
| 7. ((core or clinical or key or complex) adj2 skill*).ti,ab. |
| 8. (profession* adj2 develop*).ti,ab. |
| 9. (audit or (qual* adj2 improv*)).ti,ab. |
| 10. fluid therapy/ |
| 11. water-electrolyte balance/ |
| 12. ((fluid* or volum*) adj3 (therap* or intravenous* or iv or infusion* or drip* or administrat*)).ti,ab. |
| 13. ((fluid* or volum*) adj3 (restor* or resuscita* or replac* or deplet* or deficien*)).ti,ab. |
| 14. (fluid* adj3 (challenge or bolus)).ti,ab. |
| 15. ((crystalloid* or colloid*) adj3 (therap* or intravenous* or iv or infusion* or drip* or administrat*)).ti,ab. |
| 16. ((fluid* or volum*) adj3 (balance* or imbalance* or manag* or maint* or loss* or status or monit* or assess* or reassess* or evaluat* or prescri* or document* or chart* or protocol or strateg* or regimen* or require* or need*)).ti,ab. |
| 17. 1 or 2 or 3 or 4 or 5 or 6 or 7 or 8 or 9 |
| 18. 10 or 11 or 12 or 13 or 14 or 15 or 16 |
| 19. Hospitals.mp. or hospital/ or (hospital* adj3 staff*).mp. |
| 20. 17 and 18 and 19 |
| 21. limit 20 to (English language and humans and yr="1994 - 2016") |

[mp=title, abstract, original title, name of substance word, subject heading word, keyword heading word, protocol supplementary concept word, rare disease supplementary concept word, unique identifier]

**A2 EMBASE Search Terms**

| 1. clinical competence/ or competence/ or professional competence/ |
| --- |
| 2. clinical practice/ or professional practice/ |
| 3. (train* or educat* or teach* or apprais* or learn*).ti,ab. |
| 4. (knowledge or attitude*).ti,ab. |
| 5. (perception* or opinion* or responsibilit*).ti,ab. |
| 6. ((core or clinical or key or complex) adj2 skill*).ti,ab. |
| 7. (profession* adj2 develop*).ti,ab. |
| 8. (audit or (qual* adj2 improv*)).ti,ab. |
| 9. fluid therapy/ |
| 10. fluid balance/ or electrolyte balance/ |
| 11. ((fluid* or volum*) adj3 (therap* or intravenous* or iv or infusion* or drip* or administrat*)).ti,ab. |
| 12. ((fluid* or volum*) adj3 (restor* or resuscita* or replac* or deplet* or deficien*)).ti,ab. |
| 13. (fluid* adj3 (challenge or bolus)).ti,ab. |
| 14. ((crystalloid* or colloid*) adj3 (therap* or intravenous* or iv or infusion* or drip* or administrat*)).ti,ab. |
| 15. 1 or 2 or 3 or 4 or 5 or 6 or 7 or 8 |
| 16. 9 or 10 or 11 or 12 or 13 or 14 |
| 17. Hospitals.mp. or hospital/ or (hospital* adj3 staff*).mp. |
| 18. 15 and 16 and 17 |
| 19. limit 18 to (human and English language and yr="1994 - 2016") |

[mp=title, abstract, heading word, drug trade name, original title, device manufacturer, drug manufacturer, device trade name, keyword]

**A3 CINAHL Search Terms**

| S1 | (MH "Intravenous Therapy+") OR (MH "Fluid Therapy+") OR (MH "Intravenous Therapy (Iowa NIC)") OR "intravenous fluid therapy" OR (MH "Fluid Intake-Output Measures") OR (MH "Administration, Intravenous") OR (MH "Risk for Fluid Volume Deficit (NANDA)") OR (MH "Fluid-Electrolyte Balance+") OR (MH "Fluid-Electrolyte Imbalance+") OR (MH "Infusions, Intravenous") OR (MH "Fluid Resuscitation") OR (MH "Intravenous Nursing") OR (MH "Fluid-Electrolyte Management (Iowa NIC)") |
| --- | --- |
| S2 | TI ( ((fluid* or volum*) n3 (therap* or intravenous* or iv or infusion* or drip* or administrat*)) ) OR AB ( ((fluid* or volum*) n3 (therap* or intravenous* or iv or infusion* or drip* or administrat*)) ) |
| S3 | TI ( ((fluid* or volum*) n3 (restor* or resuscita* or replac* or deplet* or deficien*)) ) OR AB ( ((fluid* or volum*) n3 (restor* or resuscita* or replac* or deplet* or deficien*)) ) |
| S4 | TI ( (fluid* n3 (challenge or bolus)) ) OR AB ( (fluid* n3 (challenge or bolus)) ) |
| S5 | TI ( ((crystalloid* or colloid*) n3 (therap* or intravenous* or iv or infusion* or drip* or administrat*)) ) OR AB ( ((crystalloid* or colloid*) n3 (therap* or intravenous* or iv or infusion* or drip* or administrat*)) ) |
| S6 | TI ( ((fluid* or volum*) n3 (balance* or imbalance* or manag* or maint* or loss* or status or monit* or assess* or reassess* or evaluat* or prescri* or document* or chart* or protocol or strateg* or regimen* or require* or need*)) ) OR AB ( ((fluid* or volum*) n3 (balance* or imbalance* or manag* or maint* or loss* or status or monit* or assess* or reassess* or evaluat* or prescri* or document* or chart* or protocol or strateg* or regimen* or require* or need*)) ) |
| S7 | (MM "Education+") OR (MH "Professional Competence") OR (MH "Clinical Competence") OR (MM "Health Knowledge and Behavior (Iowa NOC) (Non-Cinahl)") OR (MM "Practice Patterns") OR (MM "Professional Practice") |
| S8 | TI ( (train* or educat* or teach* or learn*) ) OR AB ( (train* or educat* or teach* or learn*) ) |
| S9 | TI ( (knowledge or attitude* or perception*) ) OR AB ( (knowledge or attitude* or perception*) ) |
| S10 | TI ( opinion* or responsibilit* ) OR AB ( opinion* or responsibilit* ) |
| S11 | TI ( core n2 skill* OR clinical n2 skill* OR prescri* n2 protocol* OR prescri* n2 practice* ) OR AB ( core n2 skill* OR clinical n2 skill* OR prescri* n2 protocol* OR prescri* n2 practice* ) |
| S12 | TI ( (core or clinical or key or complex) n2 skill* ) OR AB ( (core or clinical or key or complex) n2 skill* ) |
| S13 | TI qual* improv* OR AB qual* improv* OR TI audit OR AB audit |
| S14 | S1 OR S2 OR S3 OR S4 OR S5 OR S6 |
| S15 | S7 OR S8 OR S9 OR S10 OR S11 OR S12 OR S13 |
| S16 | S14 AND S15 |
| S17 | TX hospital* |
| S18 | S16 AND S17 (Limiters - Abstract Available; Publication Year: 1994-2016; English Language; Exclude MEDLINE records) |

**A4 SCOPUS Search Terms**

( ( TITLE-ABS-KEY ( ( fluid*  OR  electrolyte* )  W/2  ( balance*  OR  imbalance*  OR  manag*  OR  maint*  OR  loss*  OR  status  OR  monit*  OR  assess*  OR  reassess*  OR  evaluat* ) )  OR  TITLE-ABS-KEY ( ( fluid*  OR  electrolyte* )  W/2  ( re-evaluat*  OR  reevaluat*  OR  prescri*  OR  document*  OR  chart*  OR  strateg*  OR  regimen*  OR  load*  OR  require*  OR  need* ) )  OR  TITLE-ABS-KEY ( ( fluid*  OR  volum*  OR  electrolyte* )  W/2  ( therap*  OR  intravenous*  OR  iv  OR  infusion*  OR  drip  OR  drips  OR  administrat* ) )  OR  TITLE-ABS-KEY ( ( fluid*  OR  volum* )  W/2  ( restor*  OR  resuscita*  OR  replac*  OR  deplet*  OR  deficien* ) )  OR  TITLE-ABS-KEY ( ( fluid*  OR  volum* )  W/2  overload* ) )  AND  SUBJAREA ( mult  OR  agri  OR  bioc  OR  immu  OR  neur  OR  phar  OR  mult  OR  medi  OR  nurs  OR  vete  OR  dent  OR  heal ) )  AND  ( ( TITLE-ABS-KEY ( train*  OR  educat*  OR  teach* )  OR  TITLE-ABS-KEY ( profession*  W/2  develop* )  OR  TITLE-ABS-KEY ( barrier*  OR  knowledge  OR  attitude* )  OR  TITLE-ABS-KEY ( perception*  OR  opinion*  OR  ignoran*  OR  unaware  OR  responsibilit* )  OR  TITLE-ABS-KEY ( ( core  OR  clinical )  W/2  skill* ) )  AND  SUBJAREA ( mult  OR  agri  OR  bioc  OR  immu  OR  neur  OR  phar  OR  mult  OR  medi  OR  nurs  OR  vete  OR  dent  OR  heal ) )  AND  ( LIMIT-TO ( LANGUAGE ,  "English" ) )  AND  ( LIMIT-TO ( PUBYEAR ,  2016 )  OR  LIMIT-TO ( PUBYEAR ,  2015 )  OR  LIMIT-TO ( PUBYEAR ,  2014 )  OR  LIMIT-TO ( PUBYEAR ,  2013 )  OR  LIMIT-TO ( PUBYEAR ,  2012 )  OR  LIMIT-TO ( PUBYEAR ,  2011 )  OR  LIMIT-TO ( PUBYEAR ,  2010 )  OR  LIMIT-TO ( PUBYEAR ,  2009 )  OR  LIMIT-TO ( PUBYEAR ,  2008 )  OR  LIMIT-TO ( PUBYEAR ,  2007 )  OR  LIMIT-TO ( PUBYEAR ,  2006 )  OR  LIMIT-TO ( PUBYEAR ,  2005 )  OR  LIMIT-TO ( PUBYEAR ,  2004 )  OR  LIMIT-TO ( PUBYEAR ,  2003 )  OR  LIMIT-TO ( PUBYEAR ,  2002 )  OR  LIMIT-TO ( PUBYEAR ,  2001 )  OR  LIMIT-TO ( PUBYEAR ,  2000 )  OR  LIMIT-TO ( PUBYEAR ,  1999 )  OR  LIMIT-TO ( PUBYEAR ,  1998 )  OR  LIMIT-TO ( PUBYEAR ,  1997 )  OR  LIMIT-TO ( PUBYEAR ,  1996 )  OR  LIMIT-TO ( PUBYEAR ,  1995 ) OR  LIMIT-TO ( PUBYEAR ,  1994 ) )

**A5 Web of Science Search Terms**

| #1 | TITLE: (((fluid* or electrolyte*) NEAR (balance* or imbalance* or manag* or maint* or loss* or status or monit* or assess* or reassess* or evaluat* or re-evaluat* or reevaluat* or prescri* or document* or chart* or strateg* or regimen* or load* or require* or need*))) |
| --- | --- |
| #2 | TITLE: (((fluid* or volum* or electrolyte*) NEAR (therap* or intravenous* or iv or infusion* or drip or drips or administrat*))) |
| #3 | TITLE: (((fluid* or volum*) NEAR (restor* or resuscita* or replac* or deplet* or deficien*))) |
| #4 | TITLE: (((fluid* or volume) NEAR overload*)) |
| #5 | #1 OR #2 OR #3 OR #4 |
| #6 | TITLE: ((train* or educat* or teach*)) |
| #7 | TITLE: ((profession* NEAR develop*)) |
| #8 | TITLE: ((barrier* or knowledge or attitude*)) |
| #9 | TITLE: ((perception* or opinion* or ignoran* or unaware or responsibilit*)) |
| #10 | TITLE: (((core or clinical) NEAR skill*)) |
| #11 | TITLE: ((audit or (quality NEAR improv*))) |
| #12 | #6 OR #7 OR #8 OR #9 OR #10 OR #11 |
| #13 | #5 AND #12 |
| #14 | #5 AND #12 Refined by: LANGUAGES: (ENGLISH) |
